# A randomized phase II study of nutritional and exercise treatment for elderly patients with advanced non-small cell lung or pancreatic cancer: the NEXTAC-TWO study protocol

**DOI:** 10.1186/s12885-019-5762-6

**Published:** 2019-05-31

**Authors:** Satoru Miura, Tateaki Naito, Shuichi Mitsunaga, Katsuhiro Omae, Keita Mori, Toshimi Inano, Teiko Yamaguchi, Noriatsu Tatematsu, Taro Okayama, Ayumu Morikawa, Takako Mouri, Hisashi Tanaka, Madoka Kimura, Hisao Imai, Takuro Mizukami, Akira Imoto, Chihiro Kondoh, Shinsuke Shiotsu, Hiroyuki Okuyama, Makoto Ueno, Toshiaki Takahashi, Tetsuya Tsuji, Hideki Aragane, Akio Inui, Takashi Higashiguchi, Koichi Takayama

**Affiliations:** 1 Department of Internal Medicine, Niigata Cancer Center Hospital, Niigata, Japan; 2 Department of Thoracic Oncology, Shizuoka Cancer Center, 1007 Shimonagakubo, Nagaizumi-cho, Sunto-gun, Shizuoka, 411-8777 Japan; 3 Department of Hepatobiliary & Pancreatic Oncology, National Cancer Center Hospital East, Kashiwa, Japan; 4 Department of Clinical Biostatistics, Graduate School of Medicine, Kyoto University, Kyoto, Japan; 5 Clinical Research Center, Shizuoka Cancer Center, Nagaizumi, Japan; 6 Division of Nutrition, Shizuoka Cancer Center, Nagaizumi, Japan; 7 Department of Health Sciences, Kyushu University Graduate School of Medical Sciences, Fukuoka, Japan; 8 Department of Rehabilitation Medicine, National Cancer Center Hospital East, Kashiwa, Japan; 9 Division of Rehabilitation Medicine, Shizuoka Cancer Center, Shizuoka, Japan; 10 Division of Nursing, Shizuoka Cancer Center, Shizuoka, Japan; 11 Department of Pulmonary Medicine, Kyoto Prefectural University of Medicine, Kyoto, Japan; 12 Department of Respiratory Medicine, Hirosaki University, Hirosaki, Japan; 13 Department of Thoracic Oncology, Osaka International Cancer Institute, Osaka, Japan; 14 Division of Respiratory Medicine, Gunma Prefectural Cancer Center, Gunma, Japan; 15 Department of Clinical Oncology, St. Marianna University School of Medicine, Miyamae-ku, Kawasaki, Japan; 16 Second Department of Internal Medicine, Osaka Medical College, Osaka, Japan; 17 Department of Medical Oncology, Toranomon Hospital, Tokyo, Japan; 18 Department of Respiratory Medicine, Japanese Red Cross Kyoto Daiichi Hospital, Kyoto, Japan; 19 Department of Clinical Oncology, Kagawa University Hospital, Kagawa, Japan; 20 Department of Gastroenterology, Kanagawa Cancer Center, Kanagawa, Japan; 21 Department of Rehabilitation Medicine, Keio University School of Medicine, Tokyo, Japan; 22 Department of Surgery, Aiseikai Yamashina Hospital, Kyoto, Japan; 23 Pharmacological Department of Herbal Medicine, Graduate School of Medical and Dental Sciences, Kagoshima University, Kagoshima, Japan; 24 Department of Surgery and Palliative Medicine, Fujita Health University School of Medicine, Aichi, Japan

**Keywords:** Cachexia, Elderly patients, Lung cancer, Pancreatic cancer, Multimodal intervention

## Abstract

**Background:**

Most advanced elderly cancer patients experience fatigue, anorexia, and declining physical function due to cancer cachexia, for which effective interventions have not been established. We performed a phase I study of a new nonpharmacological multimodal intervention called the nutritional and exercise treatment for advanced cancer (NEXTAC) program and reported the excellent feasibility of and compliance with this program in elderly patients with advanced cancer who were at risk for cancer cachexia. We report here the background, hypothesis, and design of the next-step multicenter, randomized phase II study to evaluate the efficacy of the program, the NEXTAC-TWO study.

**Methods:**

Patients with chemo-naïve advanced non-small cell lung cancer or pancreatic cancer, age ≥ 70 years, performance status ≤2, with adequate organ function and without disability according to the modified Katz index will be eligible. In total, 130 participants will be recruited from 15 Japanese institutions and will be randomized into either the intervention group or a control group. Computer-generated random numbers are allocated to each participant. Stratification factors include performance status (0 to 1 vs. 2), site of primary cancer (lung vs. pancreas), stage (III vs. IV), and type of chemotherapy (cytotoxic vs. others). Interventions and assessment will be performed 4 times every 4 ± 2 weeks from the date of randomization. Interventions will consist of nutritional counseling, nutritional supplements (rich in branched-chain amino acids), and a home-based exercise program. The exercise program will include low-intensity daily muscle training and lifestyle education to promote physical activity. The primary endpoint is disability-free survival. It is defined as the period from the date of randomization to the date of developing disability or death due to any cause. This trial also plans to evaluate the improvements in nutritional status, physical condition, quality of life, activities of daily living, overall survival, and safety as secondary endpoints. Enrollment began in August 2017. The study results will demonstrate the efficacy of multimodal interventions for elderly cancer patients and their application for the maintenance of physical and nutritional conditions in patients with cancer cachexia. This work is supported by a grant-in-aid from the Japan Agency for Medical Research and Development.

**Discussion:**

This is the first randomized trial to evaluate the efficacy and safety of a multimodal intervention specific for elderly patients with advanced cancer.

**Trial registration:**

Registered at August 23, 2017. Registry number: UMIN000028801.

**Electronic supplementary material:**

The online version of this article (10.1186/s12885-019-5762-6) contains supplementary material, which is available to authorized users.

## Background

Advances in cancer therapy have been extended not only to combinations of cytotoxic chemotherapy but also to immunotherapy using immune checkpoint inhibitors or driver-based precision medicine. These developments will provide long-term survival to many refractory cancer patients. Improving tolerability of cancer therapy and maintaining quality of life for cancer patients is thought to prolong the healthy life expectancy in patients undergoing prolonged cancer treatment. Most refractory cancer patients suffer from weight loss, fatigue, anorexia and declining physical function due to inadequate catabolism with systemic inflammation. This multifactorial syndrome is defined as cancer cachexia and is characterized by the progressive loss of body weight and reduction in skeletal muscle mass [1, 2]. The incidence of cancer cachexia was estimated to be 50–80% in patients with advanced cancer [3]. In the Japanese lung cancer population, the incidence of cancer cachexia is approximately 50% in those with advanced lung cancer, and body weight loss was related to deteriorated quality of life (QOL) and shortened survival time, regardless of the type of chemotherapy [4, 5]. However, treatment for cancer cachexia has not been well established. A prospective trial to investigate adequate interventions for cancer cachexia patients is urgently needed.

### Standard of care for cancer cachexia

Weight loss is frequently observed in advanced-stage cancer patients not only in the terminal phase but also in the early phase after diagnosis. In the terminal phase, most cachexia patients enter a stage of refractory cachexia, and they are resistant to any type of intervention. Although it is recommended that therapeutic interventions be initiated as soon as possible, there is little evidence concerning the efficacy and safety of early supportive interventions for cachexia in cancer patients.

There are no approved pharmacological interventions for cancer cachexia. On the other hand, some evidence has been reported regarding nonpharmacological interventions for cancer cachexia [6]. A meta-analysis suggested that oral nutritional interventions might be effective for increasing nutritional intake and improving QOL in cachexia patients [7, 8]. A prospective randomized phase II study was conducted to evaluate the efficacy of dietary advice in patients with cachexia [9]. This trial suggested that early dietary advice might be effective at increasing food intake. On the other hand, physical exercise has the potential to improve physical function in cancer patients undergoing chemotherapy [10]. However, these types of unimodal interventions do not improve mortality. Multimodal interventions might be required to improve the mortality rate of elderly patients with cachexia because it is a multifactorial syndrome. Notably, the current consensus report recommends nutritional support with physical exercise interventions as the standard of care for weight loss in cancer patients. Several studies have reported the effectiveness of the combination of nutritional interventions and exercise to maintain physical function and nutrition in patients with cachexia [11, 12]. However, prospective studies on appropriate nutritional support and exercise intensity and content are still inadequate to act on this evidence in clinical practice.

### Cancer cachexia in elderly cancer patients

The population of elderly cancer patients has been increasing due to a general increase in life expectancy. According to the Surveillance, Epidemiology, and End Results database, more than half of those diagnosed with lung cancer were people older than 70 years [13]. Elderly patients already have reduced physiological skeletal muscle and nutritional status due to aging, resulting in many elderly patients being diagnosed with cancer cachexia. We previously reported that cancer cachexia in elderly patients is strongly associated with decreased activity of daily living (ADL) scores and longer hospitalizations [14]. Furthermore, skeletal muscle depletion during chemotherapy has a negative impact on physical function in elderly non-small cell lung cancer (NSCLC) patients [15]. Because elderly patients independently have a high risk of falls and fractures, careful intervention is necessary for elderly patients with body weight loss and weak skeletal muscle. It is well known that elderly cancer patients are at risk for malnutrition, including cachexia, and they are very fragile when performing daily activities. Therefore, we selected elderly patients with refractory cancer as the participants in this study to investigate multimodal interventions for cachexia, including exercise and nutritional intervention.

### Results from early-phase prospective trial: the NEXTAC-ONE study

We reported a phase I study to evaluate the feasibility of multimodal interventions, including exercise and nutritional intervention, for elderly patients with advanced NSCLC or pancreatic cancer [16]. That study was named the “NEXTAC (Nutrition and Exercise Treatment for Advanced Cancer)-ONE” study and was performed as a prospective single-arm study to assess the feasibility of early nonpharmacological, multimodal interventions (NEXTAC program). The NEXTAC program was designed specifically for elderly cancer patients with high cachectic potential, and it consists of nutritional sessions, exercise educational sessions (muscle training and physical activity), lifestyle consultations and daily oral nutritional supplements (Inner Power®) that were implemented over an 8-week study period. We hypothesized that it is possible to initiate the NEXTAC program in the early treatment phase in elderly patients with advanced cancer, and we conducted the phase I study. The primary endpoint of the study was the participation rate, which was measured at three points during the 8-week study period. The three assessment points were as follows: at the time of study entry and 4 ± 2 weeks and 8 ± 2 weeks after the intervention started. In addition to the participation rate in the NEXTAC program, the physical activity level of each participant was evaluated using an accelerometer. The primary endpoint of the NEXTAC-ONE study was feasibility, which was defined as the proportion of participants attending ≥4 of 6 sessions. In total, 6 pancreatic cancer patients and 24 NSCLC patients were enrolled. The median age was 75 (range, 70–84) years. In total, 29 patients participated in more than 4 of the 6 sessions (96.7, 95% confidence interval; 83.3–99.4%) and did not experience serious adverse events related to these interventions. This phase I study met the primary endpoint, and we showed a high attendance rate and satisfactory safety profile for the NEXTAC program, with high compliance rates with regard to consuming the supplements, completing the diaries, and wearing the pedometers/accelerometers. Additionally, the results suggested that the NEXTAC interventions might induce behavioral changes, with consequent improvement in the patients’ QOL [17].

### Aim of the NEXTAC-TWO study

Based on the results of the NEXTAC-ONE study, we hypothesize that early nutritional and exercise interventions with lifestyle consultations will be feasible and will help maintain physical activity levels in elderly patients with advanced cancer who are at high risk of cachexia. We are conducting a randomized trial to elucidate whether the nonpharmacological, multimodal intervention (NEXTAC program) prolongs survival without disability in elderly patients with advanced NSCLC or pancreatic cancer.

The target population of this study is elderly cancer patients. We previously reported that compared with patients without cachexia, cachexic patients have an increased risk of prolonged hospital stays and higher medical costs [14]. It is well known that elderly patients are at high risk for cachexia. The prevention of the progression of cachexia in elderly patients has the potential to not only improve the QOL but also to reduce their medical expenses. There are several established nonpharmacological interventions for community-dwelling elderly populations that are designed to prevent sarcopenia, disability, or falls [18, 19]. This study will be helpful in designing new strategies to prevent cachexia in elderly cancer patients.

### Translational research of the NEXTAC-TWO study

We are also conducting a translational study to investigate new biomarkers for the diagnosis of cachexia. There is increasing evidence of a close link between cancer cachexia and plasma free amino acid (PFAA) or ghrelin levels [20, 21]. Cancer cachexia is characterized by skeletal muscle loss and anorexia [22]. Low levels of PFAAs reflect the depletion of the protein reserve by cachexia-induced skeletal muscle loss in a murine cachexia model using human pancreatic cancer cells. A low active ghrelin ratio is related to severe appetite loss in patients with advanced pancreatic cancer. This translational research aims to characterize PFAAs and plasma ghrelin levels in cancer cachexia patients.

## Methods

### Study design and participants

The NEXTAC-TWO study is a multicenter, randomized phase II study with 130 participants allocated 1:1 to an intervention group or a control group. One hundred thirty patients will be recruited from 15 Japanese institutions according to the inclusion criteria and exclusion criteria listed in Table 1. Participating institutions include public hospitals, university hospitals, and cancer centers in Japan. The list of study institutions is available in Additional file 2. Recruitment began in August 2017 and is expected to be completed in July 2020.

**Table 1:** Inclusion and exclusion criteria

Inclusion Criteria	
1) Diagnosis of advanced non-small cell lung cancer or inoperable pancreatic cancer	
2) Scheduled for systemic cancer therapy (i.e. chemotherapy, and/or targeted therapy and/or immunotherapy)	
i. In pancreatic cancer patients, planned systemic chemotherapy only allows of the combination of gemcitabine with nanoparticle paclitaxel.	
3) Eastern Cooperative Oncology Group (ECOG) performance status 0 to 2	
4) Age ≥ 70 years	
5) Body weight of 6 month ago can be confirmed	
6) An expected survival of ≥3 months as judged by investigators	
7) Written informed consent	
8) Adequate organ function	
i. Hemoglobin ≥8.0 g/dl	
ii. Total bilirubin ≤2.0 mg/dl	
iii. Aspartate aminotransferase ≤150 IU/L	
iv. Alanine aminotransferase ≤150 IU/L	
v. Serum creatinine ≤2.0 mg/dl	
vi. Arterial oxygen saturation (SpO_2_) ≥ 90% or partial pressure of oxygen in arterial blood (PaO_2_) ≥ 60 Torr in room air	
vii. Resting heart rate < 120 beat per minute	
Exclusion criteria	
1) Patients received prior chemotherapy except adjuvant chemotherapy	
2) Active malignancy requiring cancer treatment including chemotherapy, radiotherapy and surgery within 3 months	
3) Patients who need nursing care at following situation (based on Katz Index)	
i. Bathing	
ii. Dressing	
iii. Transferring	
iv. Feeding	
4) Symptomatic brain metastasis or leptomeningeal carcinoma	
5) Bone metastasis with high risk of fracture	
6) Patients who are difficult to evaluate or intervene with cardiovascular disease, bone and joint disease, neuromuscular disease etc.	
7) Patients who are difficult to participant due to psychiatric disorder	
8) Patients who are difficult to oral intake	
9) History of acute myocardial infarction, unstable angina, uncontrollable hypertension, and symptomatic sustained arrhythmia within 6 months. Stable atrial fibrillation treated with appropriate anticoagulation therapy is allowed.	
10) Uncontrollable diabetes mellitus	
11) Patients whose appropriate period has not passed since prior treatment	
i. Surgery: 7 days	
ii. Palliative radiation: 1 day	
iii. Minor surgery (i.e. biopsy): 1 day	
12) Screening failure	
i. Patients who failed to wear the accelerometer for more than 4 days	
ii. Patients who could not properly handle the accelerometer	
iii. Patients who failed to initiate first-line therapy within 14 days of enrollment	

**Fig. 1:**
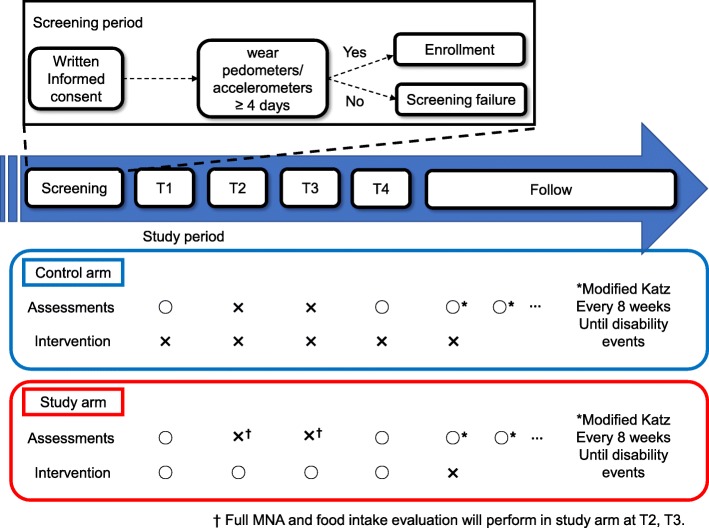
Study Schema: this study is consisted of two periods; screening period and study period

This study consisted of two periods, namely, the screening period and the study period (Fig. 1). In the screening period, after written informed consent is obtained, the investigator educates the eligible patients regarding the wearing and handling of an accelerometer. Patients who demonstrated their ability to wear and handle the accelerometer for at least 4 days are enrolled and randomized. If the patients cannot wear the accelerometer or the investigator determines that the patient is not able to handle the accelerometer adequately, the patient is judged to be ineligible and is not enrolled in the study. The study period is defined as the time between the date of study enrollment and the date of the occurring disability event or death.

### Assessments and interventions

The details of the assessment and exercise program in the NEXTAC-TWO study are shown in Table 2. There are no restrictions in concomitant care or interventions in both intervention and control group. No provision for ancillary or post-trial care is planned.

**Table 2:** Assessments and interventions

			Control arm			Intervention arm		
Sessions		Time point^a^	T1	T2/T3	T4	T1	T2/T3	T4
		Detail of assessment/intervention						
Nutritional sessions 30 min at T1 20 min at T2, T3, and T4	Assessments	•Food intake	○	–	○	○	○	○
		• Nutritional status (MNA^d^)	○	–	○	○	○	○
		• Nutritional checklist^b^	○	–	○	○	○	○
		•Skeletal muscle analysis	○	–	○	○	–	○
		•Diet diary collection	–	–	–	–	○	○
	Interventions	• Nutritional advice	–	–	–	○	○	○
		• Support for NIS^d^ management, food environment, and eating-related distress	–	–	–	○	○	○
		• ONS^d^ prescription^c^	–	–	–	○	○	–
Exercise session 30 min at T1 20 min at T2, T3, and T4 each for A and B.	Assessments	• Assessment of physical function (Hand-grip strength, SPPB^d^)	○	–	○	○	–	○
		• Exercise diary collection	–	–	–	–	○	○
		• Assessment of physical activity (Physical activity measurement, Physical activity interview)	○	–	–	○	○	○
		• EORTC-QLQ-C30 questionnaire	○	–	○	○	–	○
	Interventions	A. Home-based resistance training						
		• Prescription (T1) and modification (T2, T3, T4) of exercise program	–	–	–	○	○	○
		• Instruction of exercise procedures	–	–	–	○	○	○
		•Education of self-modification	–	–	–	○	○	○
		B. Physical activity promotive counseling						
		• Prescription of target daily step	–	–	–	○	○	○
		• Physical activity counseling	–	–	–	○	○	○
		• Education of fall prevention	–	–	–	○	○	○

^a^Baseline assessments were performed during the time between study entry and initiation of chemotherapy (T1 point). Subsequent assessments were at 4 ± 2 (T2 point), 8 ± 2 (T3 point), 12 ± 2 (T4 point) weeks after the study enrollment

^b^Nutritional checklist to collects information about presence or absence of nutrition impact symptoms, problems in food environment, or eating-related distress

^c^A branched-chain amino-acids rich oral nutritional supplement (Inner Power®, Otsuka Pharmaceutical Co., Ltd., Japan) was provided one pack daily for 12 weeks

^d^*MNA* Mini-nutritional assessment, *NIS* nutrition impact symptoms, *ONS* oral nutritional supplement, *SPPB* Short Physical Performance Battery

#### Time points of assessments and interventions

Exercise and nutritional assessment data are collected at four time points during the study period; baseline assessments are performed during the study entry until the administration of the first systemic therapeutic measure (T1 point). Systematic therapy includes cytotoxic chemotherapy, immunotherapy using an immune checkpoint inhibitor or targeted therapy using a tyrosine kinase inhibitor. These factors include stratification factors (cytotoxic chemotherapy or immunotherapy/targeted therapy). Subsequent assessment points occur at 4 ± 2 (T2 point), 8 ± 2 (T3 point) and 12 ± 2 (T4 point) weeks after randomization. After the T4 point, follow-up assessments including the determination of the modified Katz index score are performed at 8 ± 4-week intervals. In the control group, exercise and nutritional assessments are performed at the T1 and T4 assessment points. In this group, interventions are not performed at any assessment point. In the intervention group, exercise and nutritional interventions are performed at the T1-T4 points.

#### Physical and exercise assessment

The physiotherapists assess the handgrip strength and short physical performance battery (SPPB) score according to the study protocol. The medical doctor, physiotherapist or nurse administers the lifestyle questionnaire in an interview and educates the participant about lifestyle measures needed to ensure their safety and to maintain the prescribed amount of physical activity. The lifestyle questionnaire includes information about the family structure, occupational status, the amount of physical activity on a usual day, routine habits and housework, participation in sports or exercise, frequency of going out, and experiences of falls. Physical activity is measured using an accelerometer (Lifecorder®, Suzuken Co. LTD., Nagoya, Japan) at the protocol-defined assessment intervals. The QOL assessments are performed at T1 and T4 using the EORTC-QLQ-C30 questionnaire.

#### Nutritional assessment

At every assessment point, body weight (kg) is measured to the nearest 0.1 kg. The registered nutritionist assesses the nutritional status using the full version of the Mini Nutritional Assessment (full MNA®) and estimated ingested nutritional quantity using a 24-h recall method.

Lumbar skeletal muscle mass is measured at T1 and T4 by analyzing electronically stored CT images using the Slice-O-matic software (version 5.0, Tomovision, Montreal, Quebec, Canada) as previously reported [23].

#### Exercise and lifestyle interventions

**Fig. 2:**
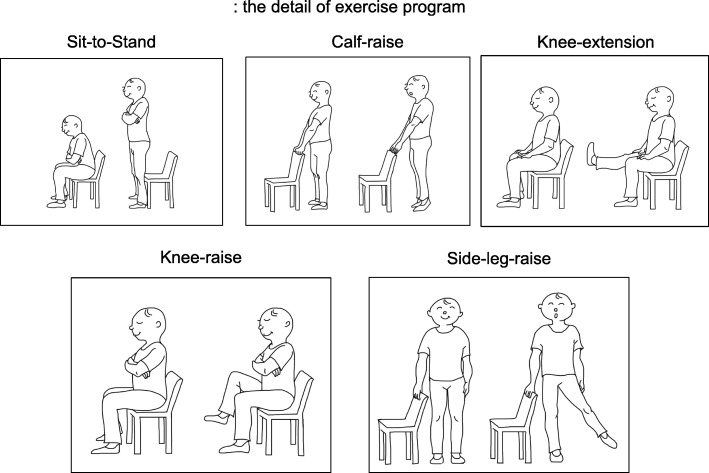
The detail of exercise program

The exercise and lifestyle interventions consist of a home-based muscle training program and physical activity program, respectively. Muscle training programs include three or five of the following five exercises: sit-to-stand, calf raise, knee extension, knee raise, and side-leg-raise with or without ankle weight (Fig. 2). One set includes 10 repetitions of each exercise. Patients perform 3 sets in a standard regimen, although that amount can be reduced based on the assessment made by the physiotherapists. Patients perform each exercise at the prescribed level three times a day at home. At T1, the physiotherapist educates the patients about level 2 of the muscle training program based on the standardized exercise education manual (Additional file 1: Appendix 1A). Physiotherapists choose the optimal level that will be followed by the patient until the next assessment according to the modified Borg scale (Additional file 1: Appendix 1B, C). Physiotherapists repeat this evaluation at each assessment point and prescribe the optimal exercise level that is to be followed until the next assessment (Additional file 1: Appendix 1D). At T4, physiotherapists prescribe the optimal exercise level that should be adhered to during the follow-up period. The physical activity program consists of lifestyle consultations and prescribed targets to encourage the patients to engage in the activities daily. The instructors in this program can be any medical staff member who understands the standardized manual (cf. medical doctor, nurses, physiotherapists, or occupational therapists). At T1, the instructor evaluates and discusses with participants the effectiveness of maintaining daily activities based on the results of the lifestyle questionnaire. If there are any issues that precludes the patient from safely implementing the lifestyle interventions, the instructor provides the participant with modifications to make the lifestyle interventions safer. The instructor prescribes individual targets for the next period according to the averages obtained previously (Additional file 1: Appendix 1E). At T4, the instructor prescribes the optimal target for the follow-up period.

#### Nutritional interventions

The nutrition intervention is mainly administered by registered nutritionists who understand the standardized manual for the NEXTAC-TWO study. The registered nutritionists assess the nutritional intake and status of the participants based on the full MNA® and estimate the ingested nutritional quantity using a 24-h recall method. They counsel the patients on how to improve their nutritional status of participants and prescribe supplements rich in branched-chain amino acids (Inner Power®, Otsuka Pharma Co., Ltd., Tokyo, Japan) for 12 weeks.

### Study objectives

The primary endpoint and secondary endpoints are listed in Table 3. The primary endpoint of this study is the disability-free survival. This endpoint is defined as the period from random assignment to the date of patients being evaluated as needing care based on the modified Katz index or death due to any cause. Overall survival is defined as the period from random assignment to the date of death due to any cause. Lean body mass (LBM) is one of the secondary endpoints. We use the lumber skeletal muscle mass index to analyze electronically stored CT images to estimate the LBM.

**Table 3:** Endpoints for the trial

Primary endpoint	
The disability-free survival	
Secondary endpoint	
1) Lean body mass assessed by CT	
2) Physical function assessed by the hand-grip strength, SPPB score and physical activity measured by accelerometer	
3) Nutritional status assessed by body weight, MNA score and ingested nutritional quantity	
4) Quality of Life assessed by EORTC-QLQ-C30	
5) The proportion of patients treated with subsequent chemotherapy	
6) Overall survival, Progression free survival	
7) The cumulative number of days of hospitalization	
8) Activity of daily life	
9) Safety	

*CT* computed tomography, *SPPB* short physical performance battery, *MNA* Mini Nutritional Assessment, *EORTC-QLQ* The European Organization for Research and Treatment of Cancer Quality-of Life Questionnaire

### Data collection and monitoring

All data related to this study is collected through the electric data capturing system within 2 weeks of each planned visit. The data center at the clinical trial management unit of Shizuoka Cancer Center, Japan, is in charge for quality control of study data. Data managers will monitor an allowance of data entry and check range for data values. The role of data monitoring committee (DMC) is to review efficacy and toxicity of study intervention independently from the investigators. The DMC review reports of severe adverse events from researchers, monitoring reports from the data center, and analysis reports from biostatisticians. The DMC comprised from three medical doctors who are independent from the study and have no conflict of interest associated with this study. No regular auditing is planned.

### Statistical considerations

We set the recruitment period to 3 years, starting in August 2017. All participants who pass the screening are randomly assigned to the intervention group or the control group at a ratio of 1:1 in every stratified group, and they are followed for 2 years after randomization. The levels of stratification factors are as follows: (1) NSCLC or pancreatic cancer, (2) PS = 0/1 or PS = 2, and (3) cytotoxic chemotherapy or not.

With 64 patients per group, the trial achieves 80% power to detect an improvement in median disability-free survival from 8 months in the control group to 12 months in the intervention group using a two-sided log-rank test with a significance level of 0.20. Because we observed only a few dropouts from the NEXTAC program in the phase I study, we estimated the needed sample size with a small number of additional patients included. In total, 130 patients will eventually be included in the study. The disability-free survival and overall survival are estimated using the Kaplan-Meier method. To compare categorical variables, chi-square or Fisher’s exact tests are used. Continuous measures are compared using the Wilcoxon rank-sum test. For the primary endpoint analyses, *p*-values < 0.05 are considered significant.

### Blood testing protocol in translational research

The blood samples are collected T1 and T4. After overnight fasting for 10 h, blood samples (10 mL) are collected in the morning before the start of anti-cancer treatment (T1) and at the end of the NEXTAC protocol (T4). The blood collection tubes contain ethylenediaminetetraacetic acid disodium salt as an anticoagulant and are immediately placed in ice water or an ice-cold cooling container for longer than 15 min. Plasma is separated by centrifugation at 1500 g and 4 °C for 15 min. One-tenth of the volume of 1 N HCL is added to the separated plasma for ghrelin analysis. Samples are stored at − 80 °C until analysis. After thawing, the plasma samples are deproteinized using acetonitrile at a final concentration of 80% before the measurement of amino acid concentrations by high-performance liquid chromatography–electrospray ionization–mass spectrometry by precolumn derivatization [24]. Plasma levels of acyl ghrelin and des-acyl ghrelin are measured using enzyme-linked immunosorbent assay kits according to the manufacturer’s instructions.

## Discussion

The NEXTAC-TWO study is a multicenter, randomized phase II study evaluating the efficacy of nutritional and exercise interventions in patients with chemo-naïve advanced NSCLC or pancreatic cancer. After the baseline assessment, the participants are randomized to either the intervention group, which receives the multimodal intervention (NEXTAC program), or to the control group, which receives standard care alone.

The primary endpoint of this study is disability-free survival. We originally defined this endpoint to detect the effectiveness of the multimodal intervention with regard to enabling the patient to maintain their activity level during cancer therapy. A similar endpoint was used as the primary endpoint in a large phase III trial that investigated the efficacy of aspirin in the elderly population [25]. It is important not only to prolong survival but also to maintain elderly patients’ ability to perform daily life activities during cancer treatment. The evidence validating the use of these types of endpoints is insufficient. We will perform this trial and try to suggest a new endpoint for future supportive care trials for cancer patients. Furthermore, this trial also plans to evaluate improvements in nutritional status, physical condition, QOL, ADL, survival and safety as secondary endpoints.

Recently, anti-cachectic agents have been developed. Anamorelin, a novel ghrelin-receptor agonist, resulted in increased LBM in the ROMANA1/RONAMA2 trial [26]. However, this agent did not result a significant benefit with regard to the handgrip strength test. Those results were reproduced in the Japanese phase II trial [27]. These results suggest that additional interventions involving anti-cachectic agents will improve motor function and maintain ADL in cachexic patients. The combination of these emerging anti-cachectic agents and nonpharmacological interventions will be the next step to improve the ADL and QOL of cachexic patients. Our NEXTAC program is a promising candidate to use in combination with anti-cachectic agents.

To the best of our knowledge, this is the first randomized trial to evaluate the efficacy and safety of a multimodal intervention for elderly cancer patients involving interprofessional collaboration.

### Status of the trial

The NEXTAC-TWO study started in August 2017 and patient recruitment is ongoing at February 26, 2019.

## Additional files

Additional file 1: Additional information for exercise prescription. Appendix 1A: the algorism of prescribe exercise program at T1 point. Appendix 1B: the level of prescribe exercise program. Appendix 1C: modified Borg scale. Appendix 1D: the algorism of prescribe exercise program at T2–4 point. Appendix 1E: the algorism of prescribe target steps. (PPTX 52 kb)

Additional file 2: A list of ethical committees. A list of ethics committees, date of approval, and reference numbers. (DOCX 15 kb)

## Data Availability

The datasets used and/or analyzed during the current study are available from the corresponding author on reasonable request.

## Abbreviations

CT: Computed tomography
EORTC-QLQ-C30: The European Organization for Research and Treatment of Cancer Quality of Life Questionnaire
MNA score: Mini Nutritional Assessment score
SPPB score: Short Physical Performance Battery score
