# Supplementary material for: A randomized phase II study of nutritional and exercise treatment for elderly patients with advanced non-small cell lung or pancreatic cancer: the NEXTAC-TWO study protocol
Source: BMC Cancer. 2019 May 31;19:528. doi: 10.1186/s12885-019-5762-6 (PMC6544995; doi:10.1186/s12885-019-5762-6)
Supplement: Supplementary file 1 — Additional information for exercise prescription. Appendix 1A: the algorism of prescribe exercise program at T1 point. Appendix 1B: the level of prescribe exercise program. Appendix 1C: modified Borg scale. Appendix 1D: the algorism of prescribe exercise program at T2–4 point. Appendix 1E: the algorism of prescribe target steps. (PPTX 52 kb) [file 12885_2019_5762_MOESM1_ESM.pptx]

## Slide 1
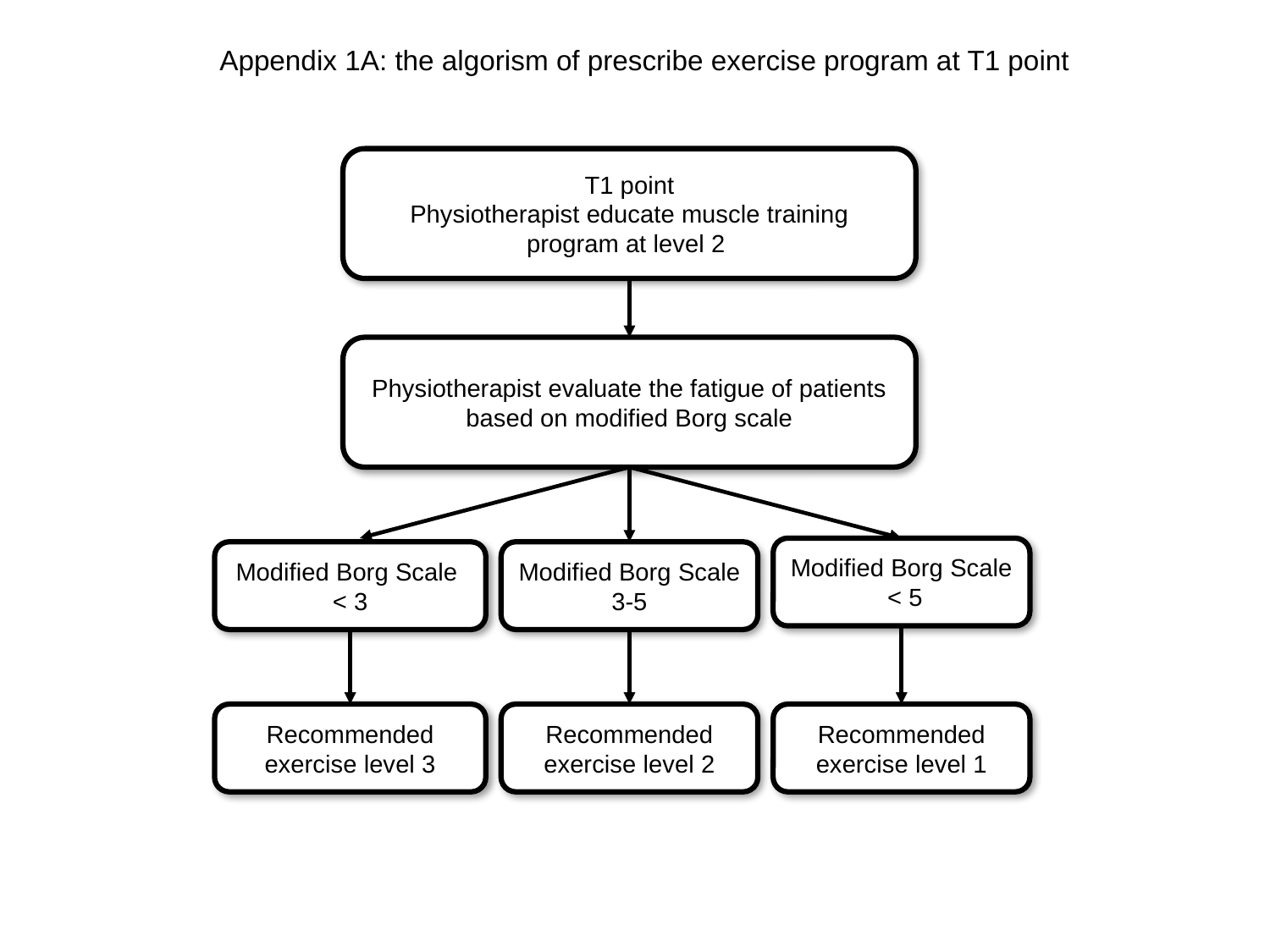

Appendix 1A: the algorism of prescribe exercise program at T1 point
T1 point
Physiotherapist educate muscle training program at level 2
Physiotherapist evaluate the fatigue of patients based on modified Borg scale
Modified Borg Scale
 < 5
Modified Borg Scale
< 3
Modified Borg Scale
3-5
Recommended exercise level 3
Recommended exercise level 2
Recommended exercise level 1

## Slide 2
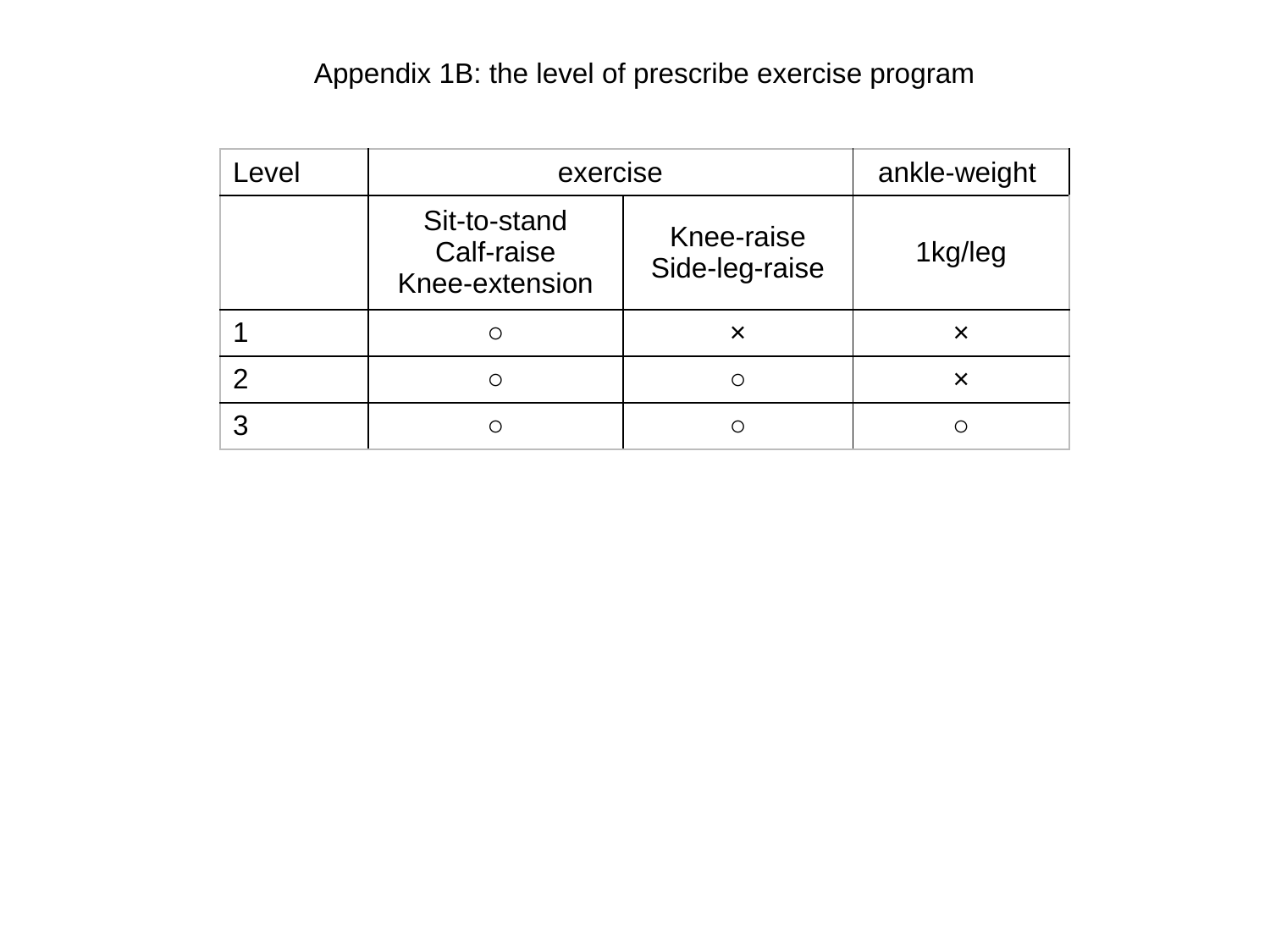

Appendix 1B: the level of prescribe exercise program
| Level | exercise | | ankle-weight |
| --- | --- | --- | --- |
| | Sit-to-stand Calf-raise Knee-extension | Knee-raise Side-leg-raise | 1kg/leg |
| 1 | ○ | × | × |
| 2 | ○ | ○ | × |
| 3 | ○ | ○ | ○ |

## Slide 3
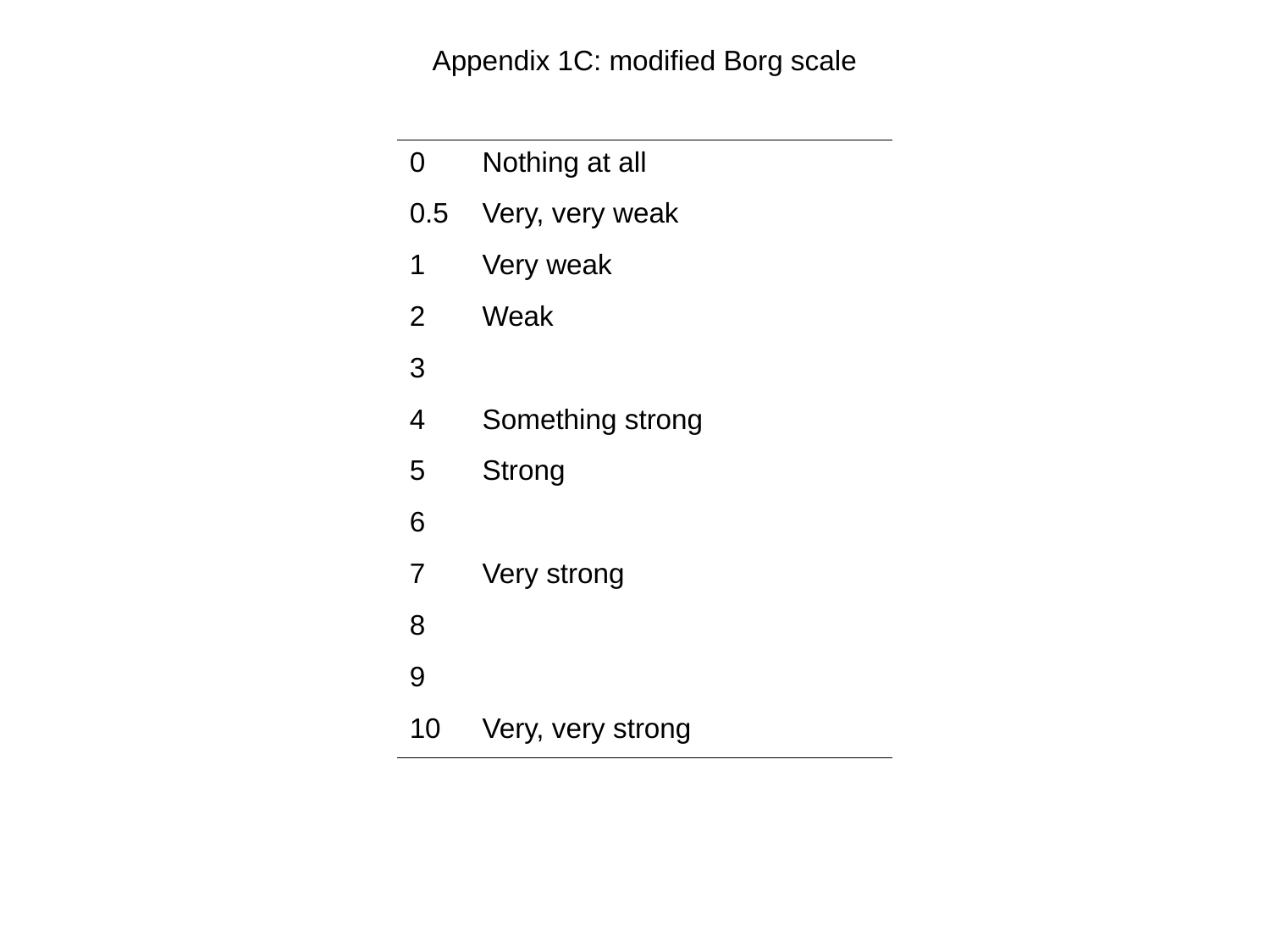

Appendix 1C: modified Borg scale
| 0 | Nothing at all |
| --- | --- |
| 0.5 | Very, very weak |
| 1 | Very weak |
| 2 | Weak |
| 3 | |
| 4 | Something strong |
| 5 | Strong |
| 6 | |
| 7 | Very strong |
| 8 | |
| 9 | |
| 10 | Very, very strong |

## Slide 4
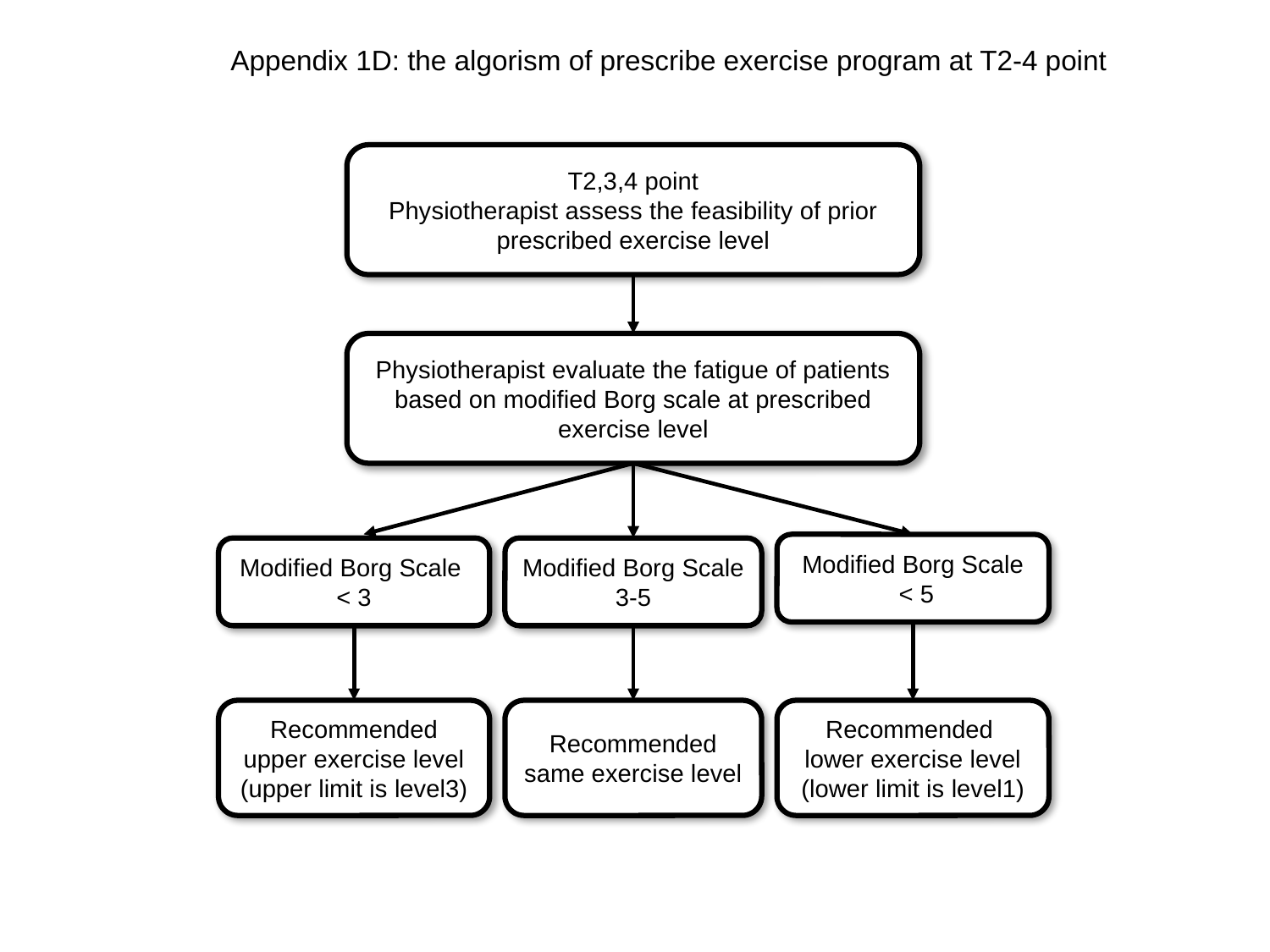

Appendix 1D: the algorism of prescribe exercise program at T2-4 point
T2,3,4 point
Physiotherapist assess the feasibility of prior prescribed exercise level
Physiotherapist evaluate the fatigue of patients based on modified Borg scale at prescribed exercise level
Modified Borg Scale
 < 5
Modified Borg Scale
< 3
Modified Borg Scale
3-5
Recommended upper exercise level
(upper limit is level3)
Recommended same exercise level
Recommended
lower exercise level
(lower limit is level1)

## Slide 5
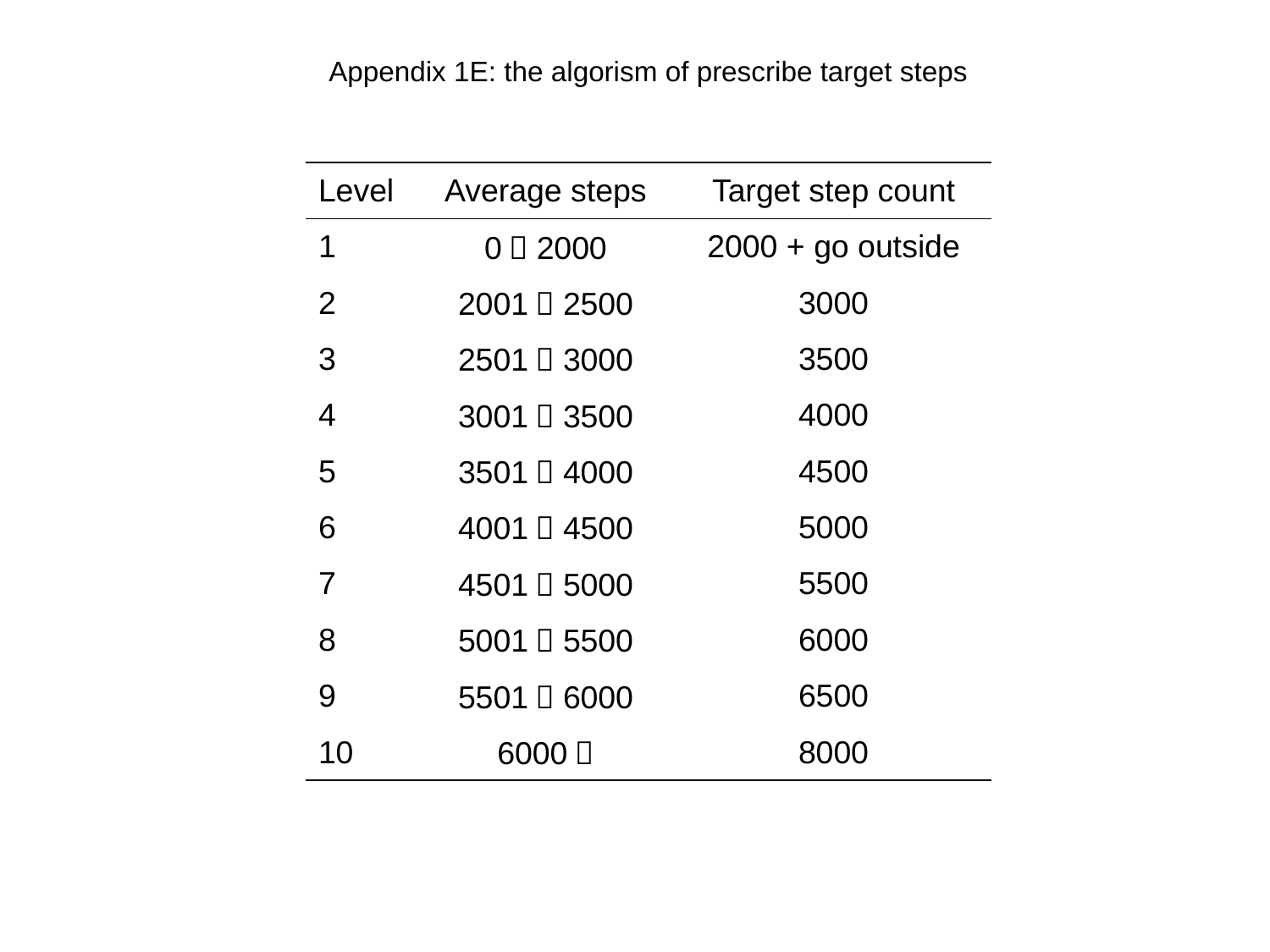

Appendix 1E: the algorism of prescribe target steps
| Level | Average steps | Target step count |
| --- | --- | --- |
| 1 | 0〜2000 | 2000 + go outside |
| 2 | 2001〜2500 | 3000 |
| 3 | 2501〜3000 | 3500 |
| 4 | 3001〜3500 | 4000 |
| 5 | 3501〜4000 | 4500 |
| 6 | 4001〜4500 | 5000 |
| 7 | 4501〜5000 | 5500 |
| 8 | 5001〜5500 | 6000 |
| 9 | 5501〜6000 | 6500 |
| 10 | 6000〜 | 8000 |
