# Supplementary material for: A randomized phase II study of nutritional and exercise treatment for elderly patients with advanced non-small cell lung or pancreatic cancer: the NEXTAC-TWO study protocol
Source: BMC Cancer. 2019 May 31;19:528. doi: 10.1186/s12885-019-5762-6 (PMC6544995; doi:10.1186/s12885-019-5762-6)
Supplement: Supplementary file 2 — A list of ethical committees. A list of ethics committees, date of approval, and reference numbers. (DOCX 15 kb) [file 12885_2019_5762_MOESM2_ESM.docx]

List of ethical committees

| The name of ethics committee | Date of approval | Reference number |
| --- | --- | --- |
| IRB of Kyoto Prefectural University of Medicine | 2017 / 12 / 12 | ERB-C-1036 |
| IRB of Niigata Cancer Center Hospital | 2017 / 9 / 29 | 2017-52 |
| IRB of National Cancer Center Hospital East | 2018 / 2 / 8 | 2017-333 |
| IRB of Shizuoka Cancer Center | 2017 / 7 / 23 | 29-12-30-1-3 |
| IRB of Osaka International Cancer Institute | 2017 / 10 / 5 | 1710059184 |
| IRB of Kinki-chuo Chest Medical Center | 2017/ 11 / 30 | 615 |
| IRB of Japanese Red Cross Kyoto Daiichi Hospital | 2017 / 10 / 2 | 642 |
| IRB of Gunma Prefectural Cancer Center | 2017 / 11 / 20 | 405-29049 |
| IRB of Toranomon Hospital | 2018 / 6 / 29 | 1557 |
| IRB of Hirosaki University | 2017 / 10 / 10 | 2017-95 |
| IRB of St. Marianna University School of Medicine | 2018 / 5 / 29 | 3881 |
| IRB of Tokyo Women's Medical University | 2018 / 10 / 2 | 181001 |
| IRB of Kanagawa Cancer Center | 2018 / 3 / 15 | 2017 Ken-69 |
| IRB of Osaka Medical College | 2017 / 12 / 13 | Rin-600(2324) |
| IRB of Kagawa University Hospital | 2017 / 11 / 24 | Heisei-29-133 |

IRB: institutional review board
